# Long-term follow-up after scleral lens fixation in patients with Marfan syndrome

**DOI:** 10.1186/s12886-017-0625-x

**Published:** 2017-12-06

**Authors:** Jan Luebke, Thomas Reinhard, Hansjuergen Agostini, Daniel Boehringer, Philipp Eberwein

**Affiliations:** Eye Center, Medical Center - University of Freiburg, Faculty of Medicine, University of Freiburg, Freiburg, Germany

**Keywords:** Marfan syndrome, Scleral lens fixation, Surgical complications, Refractive and visual outcomes

## Abstract

**Background:**

The dislocation of the crystalline lens is a common finding in patients with Marfan syndrome (MFS). Scleral intraocular lens (IOL) fixation is an accepted treatment method of this complication. To now, no long-term data on scleral IOL fixation in MFS exist.

**Methods:**

We present a retrospective study of 27 eyes of 17 MFS patients that underwent scleral lens fixation at our clinic between 1999 and 2012. These patients are compared to an age- and surgeon-matched group of 31 eyes of 27 patients who underwent the same procedure for reasons other than MFS.

**Results:**

The median age in the MFS group was 35.4 years versus 35.6 years in the non-MFS group. The median follow-up was 4 years for MFS and 3 years for non-MFS. In the MFS group, significantly more IOL-dislocations occurred than compared to the non-MFS group (30% vs. 6.5%, *p* = 0.02). Retinal detachment occurred in four MFS-eyes compared to three eyes in the non-MFS group. Biometry prediction error was 1.11 diopters (D) for MFS and 1.33 D for non-MFS (*p* = 0.11). Median BCVA (best-corrected visual acuity, logMAR) was 0.1 in the MFS group versus 0.3 in non-MFS patients.

**Conclusion:**

Scleral lens fixation in MFS patients achieves satisfying visual and refractive outcomes. Our data shows a significantly higher rate of IOL dislocations in patients with MFS. We therefore recommend addressing this complication preoperatively.

**Electronic supplementary material:**

The online version of this article (10.1186/s12886-017-0625-x) contains supplementary material, which is available to authorized users.

## Background

Marfan syndrome is a rare genetic disorder of the connective tissue, with an incidence of one to two per 10.000 individuals [1, 2]. It was first described by the French pediatrician Antoine-Bernard Marfan in 1896 [3]. Pathogenetically, mutations in the FBN1 gene—which encodes for fibrillin, a protein with crucial importance for the stability of the connective tissue—account for the disease [4, 5]. MFS affects the cardiovascular, skeletal, and ocular system with high clinical variability [6]. While cardiovascular complications, such as aortic aneurysm and mitral valve prolapse, are life threatening, abnormalities in the skeletal system, such as long and thin extremities with spidery fingers and toes, account for the typical clinical picture of MFS [2, 7]. Concerning the ocular system, typical clinical findings include an increased axial length of the eye bulb, a flattened corneal curvature, iris transillumination, early cataract, glaucoma, amblyopia, retinal detachment, and ectopia lentis or lens dislocation [8–10]. The latter occurs in about 60% of patients suffering from MFS [11, 12]. This common complication usually needs surgical treatment. In most cases, an in the bag IOL placement is not possible due to an abnormal and instable fibrillin in the lens capsule and zonular fibers [13]. In such cases scleral fixation of the IOL represents a feasible and widespread alternative [14–16].

To our knowledge, no study to date has investigated the long-term visual/refractive outcome and possible complications after scleral lens fixation in patients with MFS. This retrospective study compares these results to a group of non-MFS-patients that underwent scleral lens fixation using the same surgical techniques and surgeons during the same time period. The primary aim of this study was to assess whether scleral IOL-fixation in MFS patients shows more and/or other complications than in a group of non-MFS patients. The secondary aim was to assess the refractive and visual acuity outcomes.

## Methods

We identified eyes that underwent scleral lens fixation carried out between 1999 and 2012 by three experienced surgeons at the Eye Center of the University of Freiburg. Preoperative biometry was performed using a partial coherence interferometer (IOL Master, Carl Zeiss AG, Oberkochen, Germany) or by ultrasound biometry. IOL calculation was done using the SRK/T formula for axial lengths between 21 and 26 mm and the Haigis formula for axial lengths exceeding this interval as long as anterior chamber depth measurements were feasible.

Eyes that underwent scleral lens fixation during the same time period by the same three surgeons for reasons other than MFS were identified and utilized as a control group. Biometry and IOL calculation were performed in the same way as in the MFS group.

Different approaches for cataract extraction were chosen depending on lens status. The surgical approach depended on whether the lens was still in the lens plane or was already dislocated in the vitreous and on whether it was soft or already contained a hardened nucleus. If the lens was still in the lens plane, a pars plana approach was chosen and the lens was removed using the vitrector in the lens plane (10 eyes). If a dislocation into the vitreous had already occurred, a pars plana approach was chosen and the lens was either removed using a vitrector or, in cases with a hardened nucleus, using a fragmatome® for endophacoemulsification (13 eyes). These eyes had a primary scleral lens fixation. Four eyes of the MFS group underwent secondary IOL fixation years after phacoemulsification and intracapsular IOL implantation due to IOL-dislocation.

In the non-MFS group, 7 eyes underwent phacoemulsification with intracapsular IOL-implantation. Primary aphakia was chosen for 9 eyes due to capsular rupture with loss of sufficient capsular support. These patients then underwent secondary scleral lens fixation either due to luxation of the capsular bag and IOL (7 eyes) or in order to correct the initial aphakia (9 eyes). The other half of patients (15 eyes) underwent lens extraction using either the vitrector (14 eyes) or a fragmatome during endophacoemulsification (1 eye) after lens dislocation into the vitreous. These eyes underwent a primary scleral lens fixation (see Table 1 for an overview of surgical approaches).

**Table 1:** Methods of cataract extraction and information on timing of lens fixation in MFS- and non-MFS group

MFS	Primary lens fixation	Endophacomemulsification/fragmatome: 13 eyes
		Vitrectome from posterior: 10 eyes
	Secondary lens fixation	Phacoemulsification and subsequent IOL plus capsular bag dislocation: 4 eyes
Non-MFS	Primary lens fixation	Vitrectome via pars plana: 14 eyes
		Endophacomemulsification/fragmatome: 1 eye
	Secondary lens fixation	Phacoemulsification and subsequent IOL plus capsular bag dislocation: 7 eyes
		Phacoemulsification and subsequent aphakia: 9 eyes

The capsular bag was entirely removed in all eyes with scleral fixation of the IOL. The IOL was fixated at three and nine o’clock through transscleral sutures approximately 2 mm peripherally to the limbus similarly to sulcus fixation. After that, sutures were stitched intrasclerally in the direction of the muscular insertions of the medial and lateral rectus muscles. 10–0 Prolene sutures were employed for scleral lens fixation in all surgeries [17].

The refractive outcome was determined as the spherical equivalent calculated from subjective refraction following an objective measurement. BCVA was determined at each follow-up examination. The last available measurement was used for statistical evaluation. The difference of the target refraction and the spherical equivalent of the final refraction were calculated to determine the absolute biometry prediction error (BPE: spherical equivalent of target refraction – spherical equivalent of achieved refraction). Decimal values for visual acuity were converted into logMAR (logarithm of the minimum angle of resolution) to calculate the median values and quartiles.

For all patients, intraoperative and/or postoperative complications were recorded during in-house follow-up examinations.

All calculations and statistics were performed with R [18]. Statistical tests were used as Kaplan-Meier survival analysis and the modified Log Rank-chi^2^ test for clustered data [19, 20]. From patients that underwent scleral lens fixation in both eyes only the right eye was included in the Kaplan-Meier survival analysis. A 5% significance level was deemed statistically significant.

## Results

We identified 27 eyes of 17 MFS patients who underwent scleral lens fixation in our hospital between 1999 and 2012.

Preoperative biometry was performed by optical biometry using the IOL Master, Zeiss in 18 eyes. Ultrasound biometry was performed in the remaining eyes (*n* = 9). IOL power was calculated using the SRK/T formula for 19 eyes and the Haigis formula for 8 eyes. IOLs used were: Zeiss CT27SF (*n* = 15), Morcher Type 66 (*n* = 6), Acri.Lyc 51 N (*n* = 3), Acri.Lyc 51LC (*n* = 2), and Alcon SA60AT (n = 1).

The non-MFS group included 31 eyes of 27 patients that underwent scleral lens fixation due to reasons other than MFS. Fourteen eyes suffered from a history of trauma (blunt bulbar trauma or penetrating injuries of the eye), 10 eyes had a history of congenital cataract and therefore underwent an extraction of the crystalline lens during early childhood. Seven eyes had a history of chronic uveitis with resulting phacodonesis. Visual acuity was partly reduced due to trauma, amblyopia (in cases with congenital cataract) and chronic ocular inflammation in eyes with uveitis. Biometry and IOL power calculations were performed in the same way as in the MFS group (IOL Master, *n* = 14; ultrasound, *n* = 17; SRK/T, *n* = 28; and Haigis, *n* = 3). IOL types used were: Zeiss CT27SF (*n* = 18), Morcher Type 66 (*n* = 9), Zeiss Acri.Lyc 51 N (n = 3), and Alcon MA60AT (n = 1).

The median age in the MFS group was 35.4 years versus 35.6 years in the non-MFS group. The median follow-up time was 48 months for MFS and 36 months for the non-MFS group. More descriptive data is shown in Table 2.

**Table 2:** Descriptive data for both groups

	MFS	Non-MFS
Number	27	31
Female	9	10
Male	18	21
Median age (years)	35.4	35.6
1st Quartile	15.3	21.1
3rd Quartile	49.6	42.2
Median follow-up (months)	48	36
1st Quartile	8	9
3rd Quartile	60	54

Total number, sex, age, and follow-up period for the group of MFS and non-MFS patients (MFS: Marfan syndrome)

### Refractive and visual outcomes

Median axial length in the MFS group was 24.1 mm versus 23.6 mm in the non-MFS group. Refraction and visual acuity values were measured at least 2 months postoperatively. The median postoperative refractive astigmatism was ˗2.0D for the MFS group, while the non-MFS group layed at ˗1.87D. BPE was 1.1D in MFS versus 1.3 in non-MFS, thus showing no statistical significance (*p* = 0.38). BCVA (glasses) was 0.1 logMAR (1st quartile, 0.01, 3rd quartile, 0.22) in the MFS versus 0.3 logMAR (1st quartile, 0.07, 3rd quartile 0.7) in the non-MFS group (Table 3). A statistical analysis of these values was not performed because of concomitant visual acuity-reducing ocular diseases in the non-MFS group.

**Table 3:** Refractive and visual outcomes for both groups

	MFS	non-MFS	*p*-value (where applicable)
	(*n* = 27)	(*n* = 31)	
Median axial length (mm)	24.1	23.6	
1st Quartile	23.4	22.7	
3rd Quartile	26.3	24.8	
Median postoperative refractive astigmatism (D)	−2.0	−1.9	
1st Quartile	−3.3	−4.5	
3rd Quartile	−1.0	−1.0	
Median BPE (D)	1.1	1.3	0.38
1st Quartile	0.6	0.7	
3rd Quartile	1.5	2.2	
Median BCVA (logMAR)	0.1	0.3	*No statistical comparison because of varying visual acuity limiting concomitant ocular diseases in the non-MFS group*

Axial length, postoperative corneal astigmatism, BPE and BCVA, including quartiles for MFS and non-MFS patients. The *p*-value for the comparison of BPE is shown as well (*BPE* biometry prediction error, *BCVA* best-corrected visual acuity, *MFS* Marfan syndrome)

### Complications

**Fig. 1:**
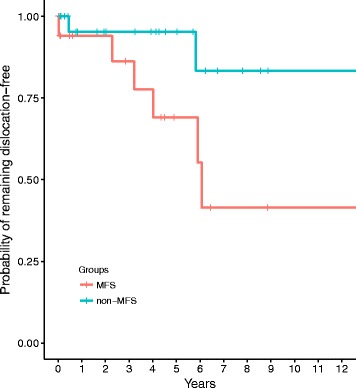
Kaplan-Meier survival analysis for MFS- (blue line) and non-MFS-group (green line). The marks on the survival curve represent the timepoints of censored data

No intraoperative complications were reported. We found no clinically significant, visual acuity-influencing, postoperative macular edema (PME, defined as visual acuity-influencing edema) in either of the two groups. Retinal detachment occurred in four cases in the MFS (14.8%) group and three cases in the non-MFS group (9.7%) (*p* = 0.1). IOL dislocation with at least one completely loosened haptic requiring a re-fixation occurred in 29.6% of the eyes in the MFS group (*n* = 8) versus 6.5% in the non-MFS group (*n* = 2) (*p* = 0.02) (Table 4). Kaplan-Meier survival analysis for both groups is shown in Fig. 1. At the median follow-up time of 48 months 22.4% of the cases in the MFS-group needed refixation while it were only 3.6% in the non-MFS group (at the median follow-up of 36 months). After 12 and 24 months each almost 6% of the eyes in the MFS group and 3.6% in the non-MFS group needed refixation. Postoperative intraocular hypotension (less than 5 mmHg) occurred in five cases of the MFS group and in three cases in the non-MFS group and lasted for a median of 2 days in both groups.

**Table 4:** Surgical complications for both groups

	MFS (n = 27)	Non-MFS (n = 31)	*p*-value (where applicable)
PME	–	–	
Retinal detachment	4 (14.8%)	3 (9.7%)	0.55
IOL dislocation	8 (29.6%)	2 (6.5%)	0.02

Total values and percentages of postoperative macular edema, retinal detachment, and IOL dislocation for the MFS and non-MFS group, including *p*-values for Pearson-chi^2^ test comparison (*PME* postoperative macular edema, *MFS* marfan syndrome, *IOL* intraocular lens)

## Discussion

This study aimed to evaluate the long-term outcome of scleral IOL fixation in MFS patients and a comparative group of patients without MFS. Our results show that both groups are comparable in regard to the refractive and visual outcomes but differ in their postoperative complications.

Groups were matched according to age and the surgeons that performed the scleral lens fixation. Non-MFS eyes had varying underlying reasons for scleral lens fixation, while the main indication for surgery in the MFS group was lens (sub)luxation. Due to concomitant retinal diseases in the non-MFS group, it is difficult to compare the visual outcomes between groups. Another typical group of patients with eyes needing a scleral lens fixation due to zonular weakness includes those having eyes with pseudoexfoliation of the crystalline lens [21]. Because these patients may show concomitant ocular pathologies limiting visual outcome, we decided to accept inhomogenous indications in the non-MFS group in favor of comparable patients’ age.

To our knowledge, no study exists that reports the outcome of scleral lens fixation in MFS patients for such a long follow-up time. Kim et al. reported on scleral lens fixation in MFS but did not compare these results to a control group and only reported on follow-up times of less than a year [22]. In both groups, a comparable refractive outcome by the biometry prediction error (1.1D in MFS vs. 1.4D in non-MFS) was found. This matches the median postoperative refractive error found by Yalniz-Akkaya et al. in a study including 96 non-MFS eyes [23]. Contrarily, two other studies by Farrahi et al. and Mutoh et al. reported a much better mean refractive error of 0.56 D and 0.1D, respectively [24, 25] but were comprised of a smaller group of patients. A major reason for this different outcome may be that we did not exclude patients with concomitant ocular pathologies such as uveitis and following ocular trauma.

Looking in more detail, our patients show a higher astigmatism in both groups (˗2D for MFS and ˗1.87D for non-MFS) than others have shown, which may account for the higher biometry prediction error in our patients [26]. It has been shown that scleral IOL fixation results in more lens tilt than regular cataract surgery with in-the-bag-placement of the IOL [27, 28]. One could well imagine that instability could be worsened in MFS eyes in general by the weakened tissue itself and by damaged ocular structures from trauma or chronic inflammatory conditions. Therefore, in our study, the refractive quality of intracapsular IOLs couldn’t be reached, and a postoperative correction with glasses or contact lenses was needed.

It is difficult to compare visual outcomes between both groups since the concomitant vision limiting ocular diseases vary broadly. Uveitis, ocular trauma, and early cataract (possibly causing amblyopia) may have a strong influence on visual acuity, while MFS itself does not affect the mere visual quality of the eye. The median best-corrected visual acuity of 0.1 logMAR in the MFS group matches the results of most other studies. Mutoh et al. reported a BCVA of 0.14 logMAR, while Taskapili et al. found a BCVA of 0.69 (decimal scale, equals logMAR of 0.15) after scleral lens fixation [25, 26].

Our results show that good visual acuity can be achieved with scleral lens fixation in MFS patients as long as there are no other visual acuity-limiting diseases.

Interestingly, no PME occurred, which accounts for one of the classical complications after cataract surgery. This statement is limited by the fact that we performed a retrospective analysis of the patients data. In our clinical experience and due to the german health care system we would expect almost every patient with a postsurgical complication to present at our clinic. In the literature, a clinically significant (defined as visual acuity-influencing edema) PME can be found after 1%–2% of cataract surgeries [29]. Angiographically, a PME can be shown in up to 30% of eyes that have undergone cataract surgery [30]. Taskapili et al. reported a PME in 10% of eyes that underwent scleral lens fixation and Yalniz-Akkaya et al. reported a PME in 6% for scleral lens fixation. Interestingly, Mutoh et al. also found 0% PME following scleral IOL fixation [23, 25, 26]. Given the rates of PME in the literature, we expected a higher rate after scleral lens fixation because of the more invasive procedure for the eye. A possible explanation for this could be the young age (35/36 years in median) of the patients in this study.

While no intraoperative complications occurred, retinal detachment was observed in both groups at higher rates than after standard extracapsular cataract extraction with intracapsular lens fixation [31]. Mahmood et al. found a retinal detachment in 1% to 4% of patients after scleral lens fixation but did not report on follow-up time in their study [32]. Other studies have reported rates of retinal detachment even after scleral lens fixation in the range of 0% to 71% but follow-up time was significantly shorter than in our group [25, 26, 31].

IOL dislocation was the most frequent complication in both groups and occurred significantly more often in the MFS group. It is difficult to find comparable reports in the literature. Most studies comprise a smaller number and a very limited follow-up time. Mutoh et al. reported one IOL dislocation out of 15 eyes with 13.3 months of follow-up, while McCluskey et al. reported one dislocation in 30 eyes during a follow-up period of 13.3 months [25, 33]. Both studies only included non-MFS eyes. Assia et al. presented two related young men who suffered from familial primary ectopic lenses. In both patients, IOL dislocations occurred after scleral lens fixation [34]. One of the possible explanations given was biodegeneration of the sutures, which might have been connected to the familial disorder that both patients shared. We do not have any evidence that could account for this theory in MFS; however, the explanation seems interesting in regard to the fact that MFS is also a genetic disorder of the connective tissue.

Postoperative hypotension occurred in a small number of eyes in both groups due to the large corneoscleral incisions that had to be made to implant the IOL. All eyes stabilized in a few days after the surgery and suffered of no longer-lasting complications and no additional surgery was needed. Mancino et al. reported on a technique based on autologous conjunctival transplantation and scleral patch graft that might help in postoperative hypotension due to wound leakage after lentectomy in MFS patients [35]. Nevertheless, in none of our patients wound leakage after the surgery occurred.

During the past few years, more surgeons have started to use iris-claw lenses to correct aphakia in eyes with insufficient capsular support. On the one hand, there is the advantage of a shorter surgery time compared to scleral lens fixation, while the refractive and visual outcome is as good as in scleral fixation [33–35]. Chronic uveitis due to fixation of the lens in the iris stroma and early corneal endothelial cell loss, at least if the IOL is positioned in the anterior chamber, may speak in favor of scleral lens fixation [36–38].

## Conclusion

In conclusion, our data suggests that scleral lens fixation in MFS is a save method and the refractive outcome after scleral lens fixation in MFS is similar to that in non-MFS patients. This conclusion might be limited by the relatively small number of included eyes.

Nonetheless, it has to be taken into account that while retinal detachment occurs equally often in MFS as in non-MFS patients, IOL dislocation is a significantly more common complication in MFS patients. We therefore recommend that this should be evaluated and discussed with MFS patients before surgery.

## Additional file

Additional file 1: The original data from which all analyses were performed. (XLSX 22 kb)

## Abbreviations

BPE: Biometry prediction error
D: Diopter
IOL: Intraocular lens
logMAR: Logarithm of the minimal angle of resolution
MFS: Marfan syndrome
PME: Postoperative macular edema
